# Vitamin C Mediates *IGFBP7* to Alleviate Chronic Atrophic Gastritis via the HIF‐1α/VEGF Pathway

**DOI:** 10.1111/jcmm.70392

**Published:** 2025-02-26

**Authors:** Xun Cheng, Hao Gu, Yulin Chong, Fan Li, Songhua Bei, Huanqing Li, Jun Jiang, Ming Pan, Li Feng, Xiaohong Zhang

**Affiliations:** ^1^ Endoscopy Center, Minhang Hospital Fudan University Shanghai China; ^2^ Department of Traditional Chinese Medicine, Minhang Hospital Fudan University Shanghai China

**Keywords:** chronic atrophic gastritis, HIF‐1α/VEGF signalling pathway, *IGFBP7*, N‐methyl‐N′‐nitro‐N‐nitroso‐guanidine, vitamin C

## Abstract

Chronic atrophic gastritis (CAG) is a precancerous lesion characterised by gastric mucosal atrophy and inflammation. Identifying key molecular mechanisms and potential therapeutic targets is essential to improve patient outcomes. Key modules and differentially expressed genes (DEGs) were recognised in the GSE153224 dataset using weighted gene co‐expression network analysis (WGCNA) and examination of differential expression. *IGFBP7* was identified as a hub gene by protein–protein interaction (PPI) network and expression validation. CAG patients’ blood parameters and gastric mucosal health status were evaluated before and after the treatment of vitamin C (VC). In addition, we investigated the effects of VC and N‐methyl‐N′‐nitro‐N‐nitrosoguanidine (MNNG) on GES‐1 cells, including cell viability, apoptosis and the expression of inflammatory and angiogenic markers. WGCNA identified that the blue module was significantly associated with CAG with a correlation coefficient 0.924. Among 93 overlapping genes, *IGFBP7* was notably underexpressed and selected as a hub gene. ROC analysis confirmed the high diagnostic performance of *IGFBP7*. CAG patients treated with VC showed significant improvement in blood parameters and improved gastric mucosal health. In vitro, VC increased cell viability, reduced cytotoxicity and apoptosis and lowered COX‐2 and apoptosis‐related protein expression in MNNG‐treated GES‐1 cells. Knockdown of *IGFBP7* further influenced these effects. MNNG upregulated HIF‐1α/VEGF signalling proteins, which VC attenuated. Combined VC and *IGFBP7* knockdown showed potential protective effects. This study highlights the regulatory role of VC and *IGFBP7* in CAG and demonstrates their potential as therapeutic targets for improving gastric mucosal health and mitigating inflammation.

## 1 Introduction

The hallmark of chronic atrophic gastritis (CAG) is persistent inflammation of the stomach mucosa, which causes the stomach glands to atrophy and be replaced by intestinal metaplasia [1]. Major aetiological factors contributing to the development of CAG include *Helicobacter pylori* infection, dietary factors, smoking and inherited tendencies [2]. The prevalence of CAG varies geographically, with higher rates reported in regions with a significant *H. pylori* infection prevalence [3]. Although CAG is generally considered a benign condition, it has been linked to a higher risk of stomach cancer. Current treatment strategies for CAG primarily focus on eradicating *H. pylori* infection, alleviating symptoms and preventing complications such as gastric ulcers and malignancy [4]. However, the efficacy of these treatments in reversing gastric atrophy and improving long‐term outcomes remains limited. Therefore, there is an urgent requirement to explore novel diagnostic biomarkers, therapeutic approaches and prognostic indicators to manage CAG better and reduce the risk of progression to GC.

Scientifically referred to as ascorbic acid, vitamin C (VC) is a water‐soluble vitamin necessary for human health [5]. As a potent antioxidant, VC scavenges free radicals and protects cells from oxidative stress [6]. In addition, VC is essential to the immune system by enhancing the efficacy of leucocytes and stimulating the production of antibodies. Research has demonstrated that VC exerts potent anti‐inflammatory effects by blocking the production of proinflammatory cytokines and molecules involved in the inflammatory cascade [7]. This dual role in supporting immune function and regulating inflammation makes VC an essential nutrient for maintaining overall health and alleviating various inflammatory diseases. Research has indicated a connection between lower gastric VC concentrations and *H. pylori* infection, CagA seropositivity and severity of gastritis. Zhang et al. found lower gastric VC levels associated with *H. pylori* infection and more severe gastritis [8]. Similarly, Yoshinaga et al. showed that VC prevented the development of gastritis in individuals with *H. pylori* infection undergoing acid suppression medication [9]. Acid suppression therapy exacerbates gastritis, but VC supplementation prevents this, indicating a protective effect against *H. pylori* ‐associated inflammation. In addition, Wang et al. showed that a combination of astaxanthin‐rich algal powder and VC reduced *H. pylori* infection and inflammation in BALB/cA mice [10]. These studies highlight the critical role of VC in regulating inflammation and maintaining overall health, especially for *H. pylori* ‐associated diseases.

Insulin‐like growth factor‐binding protein 7 (*IGFBP7*) is a regulator of insulin‐like growth factors (IGFs), influencing cell growth, differentiation and apoptosis [11]. In the context of CAG, *IGFBP7* may affect the development and progression of gastritis by regulating these processes. Emerging evidence indicates that *IGFBP7* is also involved in inflammatory processes, modulating various signalling pathways and cytokine production. Numerous studies have emphasised the significance of *IGFBP7* in the context of inflammation and renal dysfunction. Zwaag et al. reported that *IGFBP7*, a marker of renal tubular stress, increased after remote ischaemic preconditioning (RIPC) in healthy volunteers [12]. However, despite RIPC‐induced renal cell‐cycle arrest markers, the systemic inflammatory response to endotoxin (LPS) was unaffected. Conversely, Yu et al. demonstrated that *IGFBP7* correlates with kidney dysfunction [13]. They showed that *IGFBP7* knockout ameliorates kidney dysfunction, inflammation and cell death in acute kidney injury [9] models by binding to *PARP1*, inhibiting its degradation and mitigating tubular injury and inflammation. *IGFBP7* may affect the inflammatory state of CAG by regulating inflammatory signalling pathways and cytokine production. Moreover, Yang et al. demonstrated that Gypenoside XLIX exerts a protective effect against AKI by inhibiting *IGFBP7*/*IGF1R*‐mediated inflammation and programmed cell death, suggesting potential avenues for treating AKI [14]. *IGFBP7* may be involved in the pathology of CAG by affecting apoptotic pathways, such as the activity of caspase family proteins. Dysregulation of apoptosis may lead to cell injury and tissue destruction, which is a key factor in the pathology of CAG. *IGFBP7* may play a role in cytoprotective and reparative mechanisms, which may be necessary for gastric mucosal injury and repair in CAG. A deeper understanding of the intricate relationship between *IGFBP7* and inflammation may provide information on the aetiology of inflammatory illnesses and possible treatment options.

CAG is closely associated with gastric mucosal inflammation, atrophy and tumour formation. In this field, the HIF‐1α/VEGF signalling pathway has been shown to play a key role in the development of CAG. Li et al. showed that regulating the HIF‐1α/VEGF signalling pathway can inhibit angiogenesis and inflammatory responses, resulting in therapeutic effects on CAG [15]. Wen et al. showed that dehydrogenohumulin (DHE) effectively attenuated gastric injury and inhibited migration and invasion of GES‐1 cells in a rat model of MNNG‐induced CAG by inhibiting the HIF‐1α/VEGF signalling pathway [16]. In addition, Li et al. found that VC effectively inhibited testosterone‐induced prostate cell proliferation, thereby preventing benign prostatic hyperplasia (BPH), a mechanism of action that involves inhibiting the expression and stabilisation of HIF‐1α [17]. Based on these findings, our study aimed to explore the roles of VC and *IGFBP7* in CAG and probe their potential connection with the HIF‐1α/VEGF signalling pathway.

The objectives of this study were to investigate new treatment strategies and clarify the molecular processes of CAG. Using an integrated bioinformatics approach, we identified key gene modules and differentially expressed genes (DEGs) associated with CAG. Clinical relevance was assessed by evaluating blood parameters and gastric mucosal health in CAG patients before and after VC treatment. In addition, the effects of vitamin C (VC) and N‐methyl‐N′‐nitro‐N‐nitrosoguanidine (MNNG) on GES‐1 cell viability, apoptosis and the expression of inflammation‐ and angiogenesis‐related markers were examined by in vitro experiments. In addition, we investigated the effects of *IGFBP7* knockdown on these cellular processes. Our results provide insights into the regulatory roles of VC and *IGFBP7* in the aetiology of CAG and highlight their potential as therapeutic objectives to improve gastric mucosal health and reduce inflammation and cellular damage in CAG patients.

## 2 Materials and Methods

### 2.1 Download and Processing of CAG‐Related Datasets

The R program was used to preliminary the microarray dataset from GSE153224 and GSE27411 that was retrieved from Gene Expression Omnibus (GEO, https://www.ncbi.nlm.nih.gov/gds/). The GSE153224 dataset includes 5 chronic nonatrophic gastritis (CNAG) samples and 5 CAG samples. The GSE27411 dataset includes 6 CAG samples and 12 control samples. DEGs were subsequently applied to the GSE153224 dataset. The GEO2R tool performed the differential analysis once the probe ID was converted to the gene symbol. The threshold standard for fold change (FC) used was set to > 2 or < 0.5, and the *p*‐value was < 0.05.

### 2.2 Weighted Gene Co‐Expression Network Analysis (WGCNA)

A comprehensive analysis of all genes in the GSE153224 dataset was performed using the WGCNA method. Precisely calibrated to *β* = 18, the soft threshold power guaranteed scale‐free topology. A topological overlap matrix (TOM) was created by converting the weighted adjacency matrix, providing a robust network connectedness measure. Hierarchical clustering was then applied to the TOM, resulting in a dendrogram where individual branches, depicted in varied colours, represented distinct gene modules. Genes with comparable expression patterns were grouped into corresponding modules according to weighted correlation coefficients. Finally, the correlation between different gene modules and the samples in the GSE153224 dataset was analysed to identify the key module.

### 2.3 Identification of Key Overlapping Genes by Comprehensive Bioinformatics Analysis of CAG

Through the bioinformatics platform (https://bioinformatics.psb.ugent.be/webtools/Venn/), a comprehensive cross‐analysis was conducted on the critical modules identified by WGCNA, upregulated DEGs from the GSE153224 dataset and downregulated DEGs from the same dataset to identify overlapping genes. To elucidate the potential protein–protein interactions (PPI) of these overlapping genes, the Search Tool for the Retrieval of Interacting Genes (STRING) database (https://string‐db.org/) was used to conduct PPI network analysis. Molecular complex detection (MCODE), maximum clique centrality (MCC) and maximum neighbourhood component (MNC) algorithms were used to analyse the generated PPI network further. The results were visualised using Cytoscape (version 3.7.1), an open‐source network visualisation program. A statistical significance assessment of the obtained results was conducted, with the relevance threshold at *p* < 0.05. Finally, a cross‐analysis of the top 10 genes presented in three topological representations was performed using the bioinformatics platform to identify key overlapping genes.

### 2.4 Expression Analysis and Clinical Diagnosis Analysis of Key Overlapping Genes

The expression of key overlapping genes in samples of GSE153224 and GSE27411 datasets was detected and visualised using the Sangerbox website (version 3.0, http://vip.sangerbox.com/home.html). Next, the receiver‐operating characteristic [1] curve was analysed using the ‘timeROC’ package in R to assess the clinical diagnostic significance of hub genes. The sensitivity (actual‐positive rate) and 1‐specificity (false‐positive rate) were evaluated at each threshold to build the ROC curve. The clinical diagnostic relevance of the hub gene correlates with its area under the curve (AUC) and 95% confidence interval (95% CI), where a greater AUC indicates a higher significance level.

### 2.5 Measurement of Serum Biomarkers in CAG Patients

This study included 28 human samples from Shanghai Minhang Central Hospital (Shanghai, China). All samples were diagnosed with CAG at least 1 year before inclusion in the study. The patients were approximately 60 years old. The study was approved by the Ethics Committee of Shanghai Minhang Central Hospital (Approval Number: 2024‐010‐018) and conducted by the Declaration of Helsinki. Pathological results were recorded at the patient's initial visit. Each patient received 5 g of VC daily for 3 consecutive months. Blood samples were collected from the 28 subjects via venipuncture before and after VC administration to measure levels of G‐17, PGI, PGII, PGI/PGII, IL‐1β, IL‐6, TGF‐β1, PI3K, PTEN and β‐actin. The blood samples were placed in plain tubes and solidified at room temperature (25°C–27°C) for at least 30 min. After the samples were solidified, the serum was separated by centrifuging at 4000 × *g* for 10 min at room temperature (25°C–27°C). After that, the serum was kept at −70°C until it was analysed. Serum levels of G‐17, PGI, PGII, IL‐1β, IL‐6, TGF‐β1, PI3K, PTEN and β‐actin were determined using enzyme‐linked immunosorbent assay (ELISA) kits. Absorbance for these ELISA‐based assays was evaluated at 450 nm using a microplate reader (BioTek, USA). The concentrations of each biomarker were calculated using standard curves generated for each assay.

### 2.6 Clinical Research for Patients

Pathological results were recorded at each patient's initial visit, followed by administering 5 g of VC daily for 3 consecutive months. Among the 28 patients, 12 underwent biopsy sampling, receiving endoscopic examination and pathological analysis to confirm the diagnosis. The endoscopic procedures were performed using lower gastrointestinal endoscopes, including models EG‐590WR, EG‐601WR, EG‐450WM5 (Fujifilm, Tokyo, Japan), PCF‐Q260AZI, CF‐H260AI, CF‐HQ290I, PCF‐Q260JI and CF‐HQ290I (Olympus, Tokyo, Japan)—biopsy procedures adhered to strict ethical guidelines, ensuring that participants' informed permission was acquired before sampling. Additionally, sample handling and storage complied with international biosafety and quality control standards, ensuring the integrity and reliability of the collected data. Throughout the study, measures were taken to protect patient privacy, and all data were analysed anonymously.

### 2.7 Cell Lines and Culture

The Shanghai Institute of Cell Biology (Shanghai, China) provided the human stomach mucosal epithelial cells (GES‐1). They were kept in Dulbecco's Modified Eagle Medium (DMEM), enhanced with 1% penicillin–streptomycin and 10% foetal bovine serum (FBS). Cell cultures were maintained at 37°C in a humidified environment with 5% CO_2_.

### 2.8 Cell Treatment and Transfection

GES‐1 cells were cultured in 6‐well plates and exposed to 10, 20, 40 and 80 μM MNNG for 0, 12, 24 and 48 h to induce CAG. Additionally, GES‐1 cells were exposed to varying VC concentrations at 100, 200 and 300 nM for 24 h. For transient transfection, a density of 2 × 10^5^ cells per well was used to seed GES‐1 cells in 24‐well plates. Specific small‐interfering RNAs (siRNAs) targeting *IGFBP7*, namely si‐*IGFBP7*‐1 and si‐*IGFBP7*‐2, as well as a negative control siRNA (si‐NC), were transfected into the cells to achieve knockdown of *IGFBP7* expression. The cells were left to culture for an appropriate duration to ensure efficient knockdown of *IGFBP7*. Transfection was performed by Lipofectamine 3000 (Invitrogen, Shanghai, China) in compliance with the manufacturer's guidelines.

### 2.9 Cell Counting Kit‐8 (CCK‐8) Assay

GES‐1 cells were planted at a density of 5 × 10^3^ cells per well in 96‐well plates. The CCK‐8 reagent was applied to each well according to the manufacturer's instructions following the specified procedures. The plates were cultivated for the specified time to allow the formation of the formazan dye. Next, using a microplate reader (Kehua Technologies Inc, Shanghai, China), absorbance was measured at 450 nm. The absorbance values were used to assess cell viability.

### 2.10 Quantitative Real‐Time Polymerase Chain Reaction (qRT‐PCR)

Following the manufacturer's instructions, the TRIzol reagent (Tiangen, Beijing, China) was used to extract the total RNA of GES‐1 cells. We utilised a PrimeScript RT kit from Dalian, China, for cDNA synthesis. To do qRT‐PCR with the StepOnePlus Real‐Time PCR System (Applied Biosystems, Shanghai, China), SYBR Green PCR Master Mix (Takara, China) was utilised. The levels of gene expression were measured and adjusted for GAPDH. The 2^−ΔΔCT^ method was used to compute each target expression level. A primer sequence was set in Table 1.

**TABLE 1:** Primer sequences for qRT‐PCR.

Target	Direction	Sequence (5′–3′)
*COX‐2*	Forward	AATCTGGCTGCGGGAACACAAC
*COX‐2*	Reverse	TGTCTGGAACAACTGCTCATCACC
*Caspase‐3*	Forward	AGCAAACCTCAGGGAAACATT
*Caspase‐3*	Reverse	CTCAGAAGCACACAAACAAAACT
*Caspase‐9*	Forward	AACCCTAGAAAACCTTACCCC
*Caspase‐9*	Reverse	CATCACCAAATCCTCCAGAAC
*IGFBP7*	Forward	CGAGCAAGGTCCTTCCATAG
*IGFBP7*	Reverse	GGTGTCGGGATTCCGATGAC
*Bcl‐2*	Forward	GCTGGACATTGGACTTCCTC
*Bcl‐2*	Reverse	GCTGGACATTGGACTTCCTC
*Bax*	Forward	GCTGGACATTGGACTTCCTC
*Bax*	Reverse	ACCACTGTGACCTGCTCCA
*HIF‐1α*	Forward	TGACCAGCAACTTGAGGAAGTACCATTAT
*HIF‐1α*	Reverse	GGTGGGTAATGGAGACATTGCCAAATTT
*VEGF*	Forward	GCCTTGCCTTGCTGCTCTACC
*VEGF*	Reverse	CTTCGTGATGATTCTGCCCTCCTC
*VEGFR2*	Forward	AGGGAGTCTGTGGCATCTGAAGG
*VEGFR2*	Reverse	GTGGTGTCTGTGTCATCGGAGTG
*Paxillin*	Forward	AACGGCCAGTGTTCTTGTCAG
*Paxillin*	Reverse	AACGGCCAGTGTTCTTGTCAG
*SRC*	Forward	TTTGGCAAGATCACTAGACGGG
*SRC*	Reverse	GAGGCAGTAGGCACCTTTTGT
*GAPDH*	Forward	ATCAATGGAAATCCCATCACCA
*GAPDH*	Reverse	GACTCCACGACGTACTCAGCG

### 2.11 Western Blot (WB) Assay

Protease and phosphatase inhibitors (CoWin Biosciences, Nanjing, China) were added to the RIPA lysis buffer (Solarbio, Beijing, China) to facilitate the preparation of protein lysates from GES‐1 cells. The BCA Protein Assay Kit (Beyotime, China) was used to measure the protein concentration. Proteins in equal quantities were separated using 10% SDS‐PAGE and then put onto PVDF membranes from Beyotime in Beijing, China. Membranes were exposed to primary (COX‐2, Caspase‐3, Caspase‐9, IGFBP7, Bcl‐2, Bax, HIF‐1α, VEGF, VEGFR2, Paxillin and SRC, Abcam, China, 1:1000) and suitable secondary antibodies after being blocked with 5% skim milk. GAPDH (Kangcheng, Shanghai, China, 1:5000) was employed as an internal reference. An enhanced chemiluminescence (ECL) kit from Tiangen in Beijing, China, was used to visualise the protein bands, and a ChemiDoc imaging equipment from Bio‐Rad in Shanghai, China, was used to take the images.

### 2.12 Lactate Dehydrogenase (LDH) Release Assay

Cellular damage in GES‐1 cells was assessed by measuring LDH published onto the culture media. After treatment with different concentrations of phosphate‐buffered saline (PBS) for 48 h, the culture media was collected by centrifugation for 10 min at 3000 × *g*. The LDH content in the collected culture medium was determined using a spectrophotometric‐based LDH assay kit (KeyGEN Biotech, China). LDH levels were expressed in milliunits per millilitre (mg/mL).

### 2.13 Flow Cytometry

GES‐1 cells were separated using trypsin–EDTA (Life Technologies, Beijing, China) for flow cytometry analysis and then cleaned with PBS. As directed by the manufacturer, stain cells with Annexin V and propidium iodide (PI) to distinguish between live, apoptotic and necrotic cells. A flow cytometer (Jiyuan, Guangzhou, China) was used for the flow cytometry, and FlowJo software (FlowJo, Hangzhou, China) was used for data analysis to calculate the cell apoptosis rate.

### 2.14 Statistical Analysis

For statistical analysis, we used R software and ensured that each experiment was repeated at least three times to enhance the reliability of the results. We reported the mean ± standard deviation (mean ± SD) for experimental results. When comparing differences between treatment groups, we primarily used one‐way ANOVA to determine the significance of the overall differences, followed by post hoc multiple comparison analyses using Tukey's test to allow for in‐depth exploration of significant differences between specific groups. A two‐sample t‐test was used to compare the two groups of data. The criterion for statistical significance was set at *p* < 0.05.

## 3 Results

### 3.1 Identify Key Modules of CAG Through WGCNA

**FIGURE 1:**
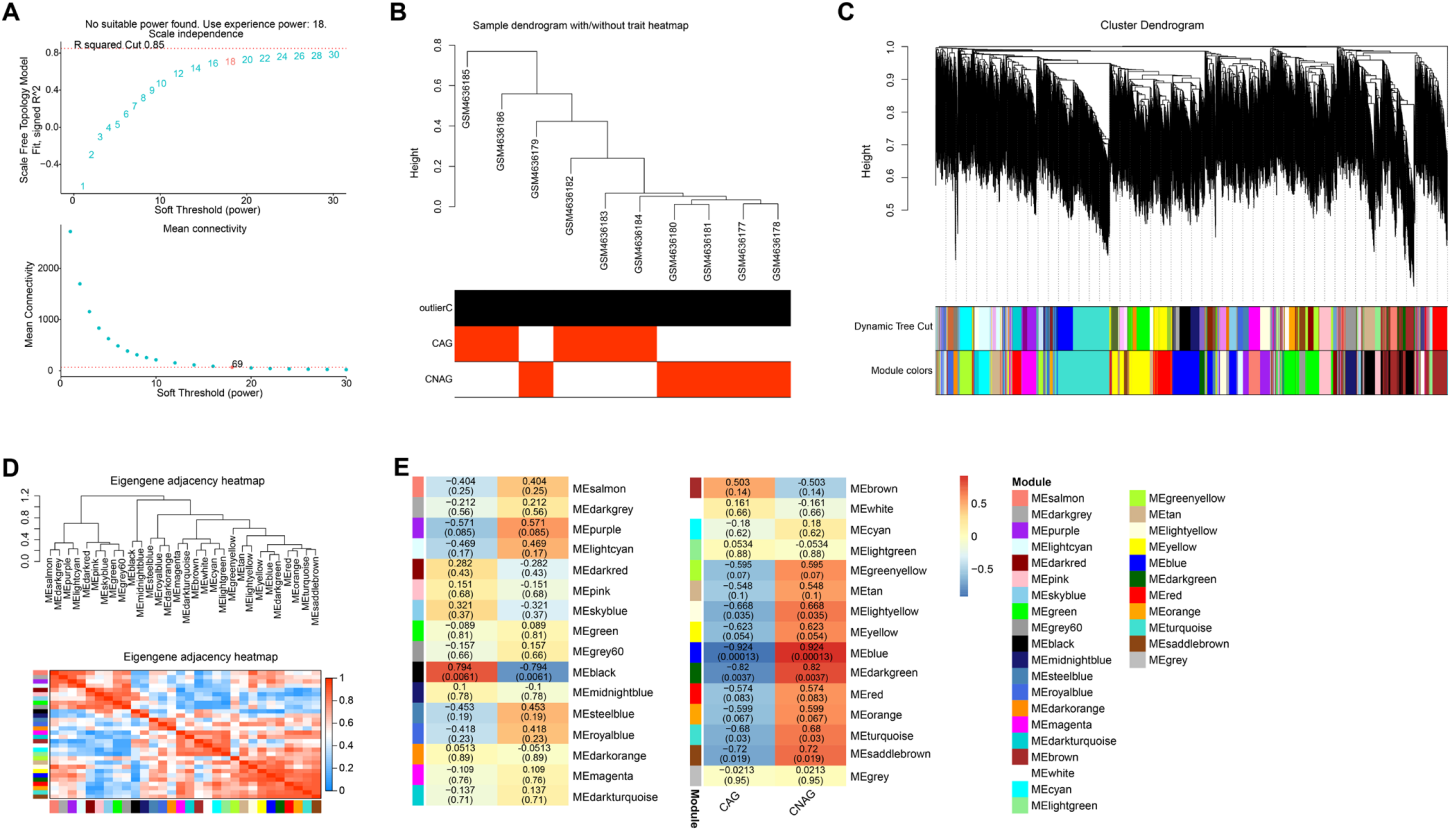
WGCNA analysis of all genes in the GSE153224 dataset. (A) Analysis of the scale‐free topology fit index (β) to select an appropriate soft threshold (power value) to construct the co‐expression network. (B) Dendrogram of genes clustered according to co‐expression patterns, with different colours representing different co‐expression modules. (C and D) The distribution of genes in modules identified in WGCNA shows the number of genes in each module. (E) Heatmap of correlations between co‐expressed modules and clinical features. Each cell contains the corresponding correlation and *p*‐value. WGCNA, weighted gene co‐expression network analysis.

As illustrated in Figure 1A, the ideal soft‐thresholding power for fitting the scale‐free topology model was determined as 18. Subsequently, an in‐depth investigation was conducted on clustering 10 samples in the GSE153224 dataset (Figure 1B). Based on the co‐expression patterns of the genes across samples, the WGCNA technique was used to cluster the genes into multiple modules, each represented by a different colour (Figure 1C,D). To evaluate the connections among these identified modules, we investigated the adjacency of characteristic genes. Remarkably, the blue module strongly correlated with sample performance, with a coefficient of 0.924 (Figure 1E). This correlation underscores the potential relevance of the blue module to the pathogenesis of CAG.

### 3.2 Identification and Analysis of DEGs in the GSE153224 Dataset

**FIGURE 2:**
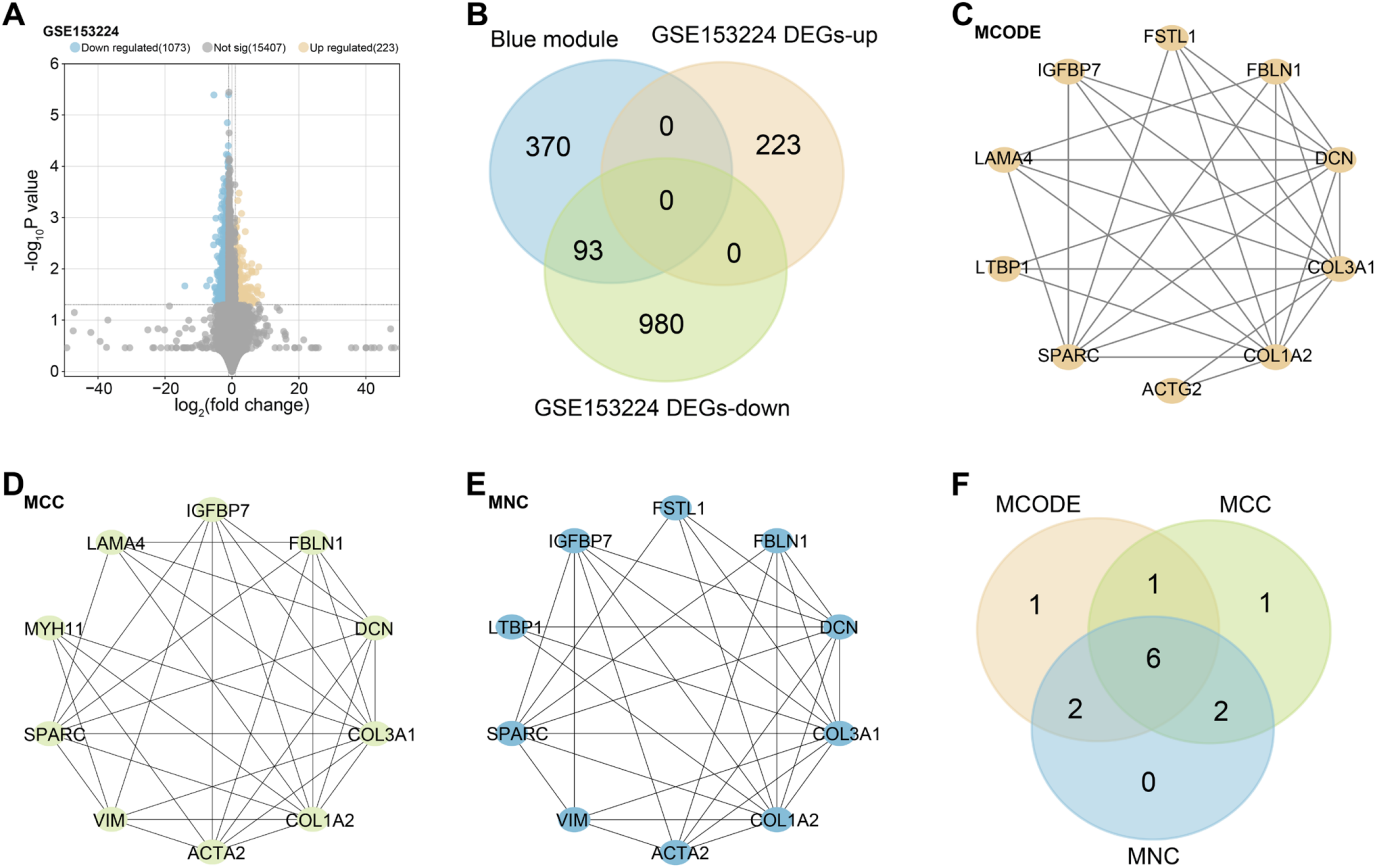
Identification and analysis of DEGs in the GSE153224 dataset. (A) Volcano plot showing the results of differential gene expression analysis, where yellow dots represent upregulated DEGs and blue dots represent downregulated DEGs. (B) Venn diagram showing the overlap of blue modules, GSE153224 upregulated DEGs and GSE153224 downregulated DEGs. (C–E) Topological analysis using the Cytoscape plugin to identify overlapping genes. (C) MCODE analysis identified 10 nodes and 28 edges. (D) MCC analysis identified 10 nodes and 33 edges. (E) MNC analysis identified 10 nodes and 31 edges. (F) Venn diagram showing the overlapping of key genes identified by three topological analyses. DEGs, differentially expressed genes; MCODE, molecular complex detection; MCC, maximal clique centrality; MNC, maximum neighbourhood component.

We identified 223 upregulated DEGs and 1073 downregulated DEGs in CAG and CNAG samples from the GSE153224 dataset (Figure 2A). Subsequently, cross‐analysis of upregulated DEGs in GSE153224, downregulated DEGs in GSE153224 and genes within the blue module resulted in 93 overlapping genes (Figure 2B). The PPI network obtained by three algorithms, MCODE, MCC and MNC algorithms, showed the networks of the top 10 genes, respectively (Figure 2C–E). Further cross‐analysis of the top ten genes in the three analyses identified 6 key overlapping genes (Figure 2F).

### 3.3 Analysis of Expression and Clinical Diagnostic Performance of Key Overlapping Genes

**FIGURE 3:**
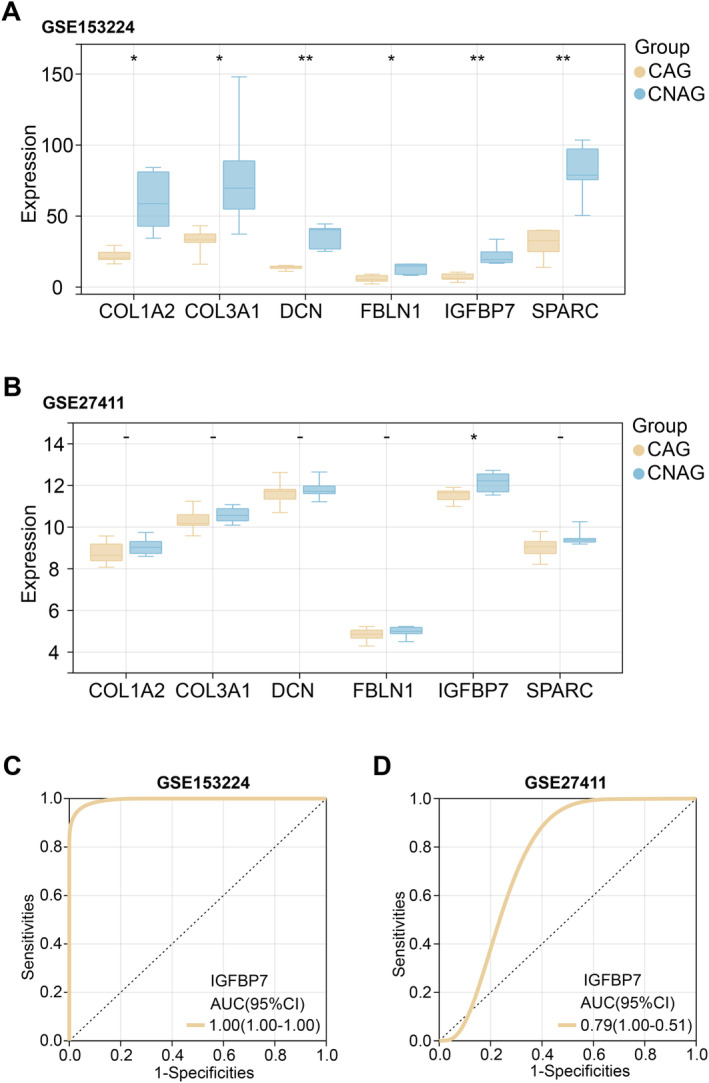
Analysis of the expression and clinical diagnostic value of key overlapping genes in CAG. (A and B) Box plots of expression analysis of 6 overlapping genes (*COL1A2*, *COL3A1*, *DCN*, *FBLN1*, *SPARC*, *IGFBP7* and *SPARC*) in different GSE153224 and GSE27411 dataset samples. Blue represents the CNAG and regular groups, and yellow represents the CAG group. (C and D) ROC curves of *IGFBP7* in GSE153224 and GSE27411 datasets. The horizontal axis represents 1‐specificity (false‐positive rate), and the vertical axis represents sensitivity (actual‐positive rate). CAG, chronic atrophic gastritis; CNAG, chronic nonatrophic gastritis; ROC, receiver‐operating characteristic. *p < 0.05, **p < 0.01.

Examination of the expression of these six key overlapping genes in the GSE153224 dataset revealed that six genes were significantly underexpressed in CAG, namely *DCN*, *SPARC*, *IGFBP7*, *FBLN1*, *COL1A2* and *COL3A1* (Figure 3A). However, in the GSE27411 dataset, only *IGFBP7* showed significant underexpression in CAG (Figure 3B). Consequently, *IGFBP7* was selected as the hub gene for this study. ROC curve analysis was done to assess the clinical diagnostic relevance of *IGFBP7*. The AUC values of *IGFBP7* in the GSE153224 and GSE27411 datasets were more significant than 0.7, indicating that the gene has a high clinical diagnostic performance (Figure 3C,D).

### 3.4 Impact of VC Treatment on Blood Parameters in CAG Patients

**FIGURE 4:**
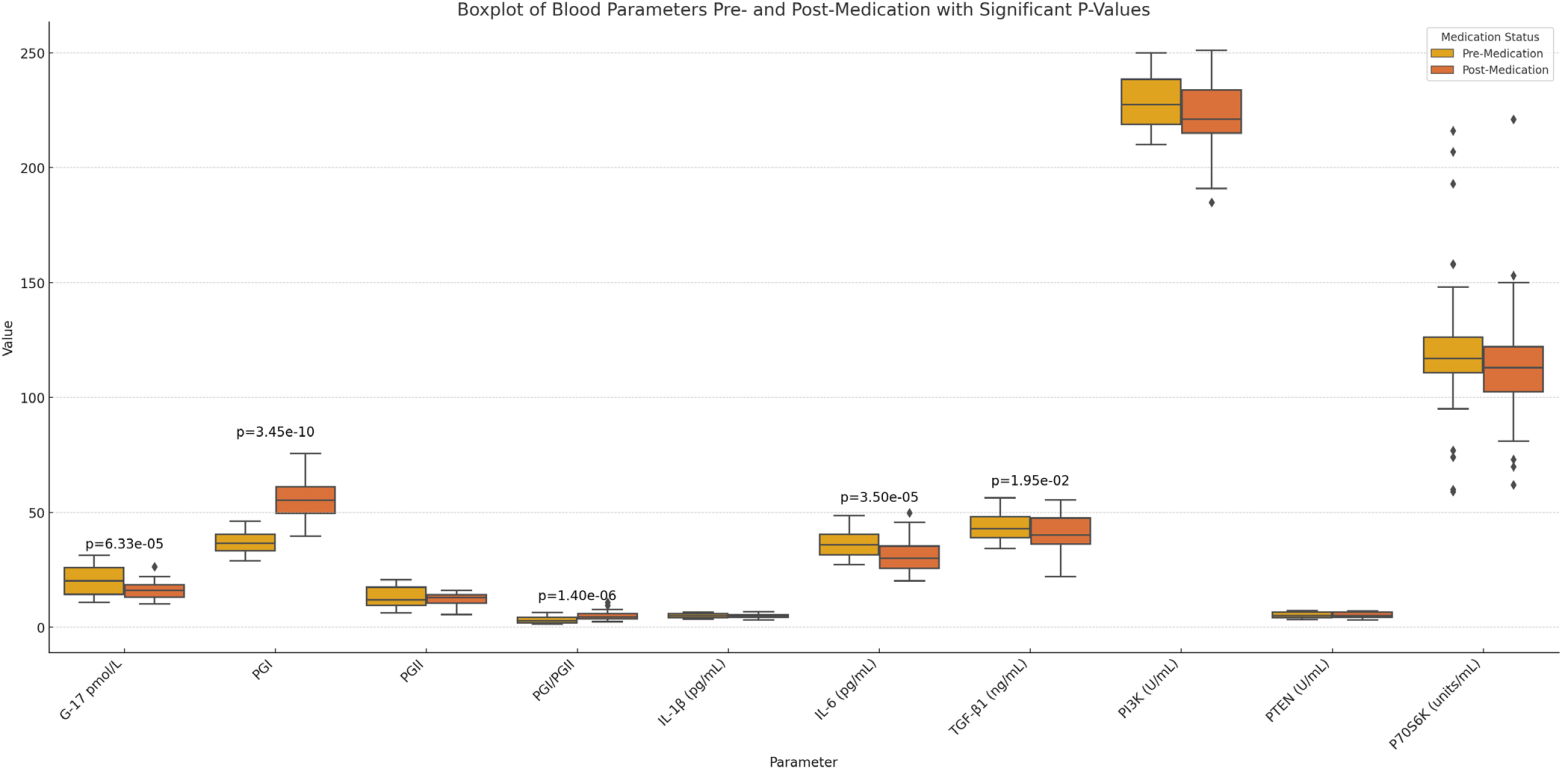
Effect of VC therapy on blood parameters in patients with CAG. Box plot analysis of the changes in specific blood parameters after VC therapy in CAG patients, including G‐17, PGI, PGII, IL‐6, TGF‐β1, PGII, IL‐1β, PI3K, PTEN and P70S6K. Yellow represents premedication, and orange represents postmedication. VC, vitamin C.

Box plot analysis (Figure 4) and supplementary data (Tables S1 and S2) revealed pronounced alterations in specific blood parameters following VC treatment in CAG patients. Gastrin‐17 (G‐17) is an essential gastrointestinal hormone closely related to gastric acid secretion and mucosal health. In patients with CAG, elevated levels of G‐17 correlate with the severity of gastric mucosal atrophy and inflammation [18]. Our study showed a significant decrease in G‐17 levels after VC treatment (*p* = 6.33e‐05), which may indicate that VC treatment helps to reduce the inflammation and atrophy of the gastric mucosa, thereby decreasing the secretion of G‐17. This change may be associated with an improvement in the condition of the patient's gastric mucosa and a reduction in the risk of disease progression. Pepsin I (PGI) is an essential indicator of gastric mucosal health and correlates with the protective mechanisms of the gastric mucosa [18]. The significant increase in PGI levels after VC treatment (*p* = 3.45e‐10) suggests that VC may enhance the defence mechanisms of the gastric mucosa, which helps to resist the damage caused by inflammation and oxidative stress. Elevated levels of PGI may be associated with gastric mucosal repair and inflammation remission, which predicts a better prognosis and a lower risk of complications. Furthermore, the PGI/PGII ratio experienced a marked elevation (*p* = 1.40e‐06), possibly reflecting improved gastric mucosal status. Regarding inflammatory and growth factors, IL‐6 levels (pg/mL) declined significantly (*p* = 3.50e‐05), highlighting VC's potential anti‐inflammatory effects, as IL‐6 is a key inflammatory marker. Meanwhile, TGF‐β1 concentrations (ng/mL) decreased significantly (*p* = 0.0195), implying a suppression of cellular proliferation and repair processes mediated by this growth factor. In contrast, VC treatment did not significantly influence other blood parameters, including PGII, IL‐1β, PI3K, PTEN and P70S6K, as evidenced by their insignificant changes before and after treatment. This observation underscores the specificity of VC's effects, primarily targeting biomarkers associated with gastrin secretion, mucosal protection, inflammation and cellular growth regulation. Of note, pepsinogen II (PGII) is a precursor of pepsin, and its levels, along with those of PGI, may reflect gastric mucosal function and inflammatory status [18]. PGII is a marker of inflammation and is influenced by several factors, including genetic susceptibility, heterogeneity of inflammatory profiles and the effect of VC on different inflammatory pathways. We hypothesised that the nonsignificant effect of VC on PGII may be related to its anti‐inflammatory mechanism. The duration of VC treatment may be a factor, suggesting that a longer duration of therapy may be required to observe significant changes in PGII levels. In addition, the dose of VC may have different effects on various parameters; the dose we used may have had little impact on PGII, or a larger dose may have been required to detect significant changes. Finally, despite our efforts to control experimental conditions, sample size and interindividual biological variation may have resulted in nonsignificant parameter changes.

### 3.5 Improved Gastric Mucosal Health After Vitamin C Treatment

**FIGURE 5:**
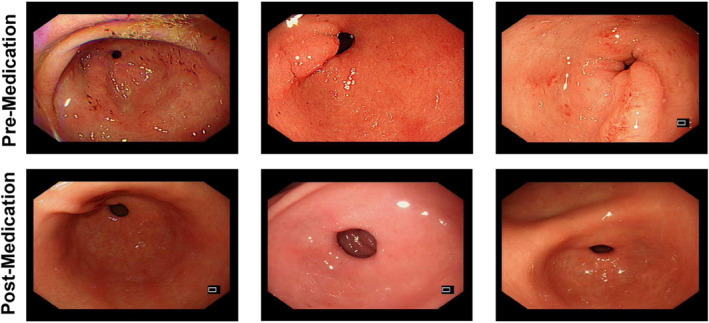
Gastric mucosa significantly improved after 3 months of VC treatment. Endoscopic images of three patients before and after 3 months of continuous vitamin C administration. The upper row shows the gastric mucosa before treatment, displaying mucosal erythema, erosion and atrophy. The lower row shows the posttreatment improvements, including reduced erythema, healed ulcers and decreased inflammation. VC, vitamin C.

Endoscopic examinations conducted before and after 3 months of continuous vitamin C administration revealed substantial improvements in gastric mucosa, as demonstrated in Figure 5A and Figure S1A. Before treatment, varying degrees of mucosal erythema, erosion and atrophy were observed. Several patients exhibited acute active inflammation, characterised by notable mucosal redness, scattered erosions, superficial ulcers and mucosal congestion and oedema. Posttreatment observations indicated a marked improvement in the overall appearance of the gastric mucosa. There was a significant reduction in mucosal erythema, healing of superficial ulcers, improvement in mucosal oedema and a noticeable decrease in inflammation. These findings suggest that 3 months of vitamin C treatment positively impacts gastric mucosal health, alleviating inflammation, promoting the healing of erosions and ulcers and enhancing mucosal integrity.

### 3.6 Effects of VC and MNNG Induction on GES‐1 Cell Viability and COX‐2 Expression

**FIGURE 6:**
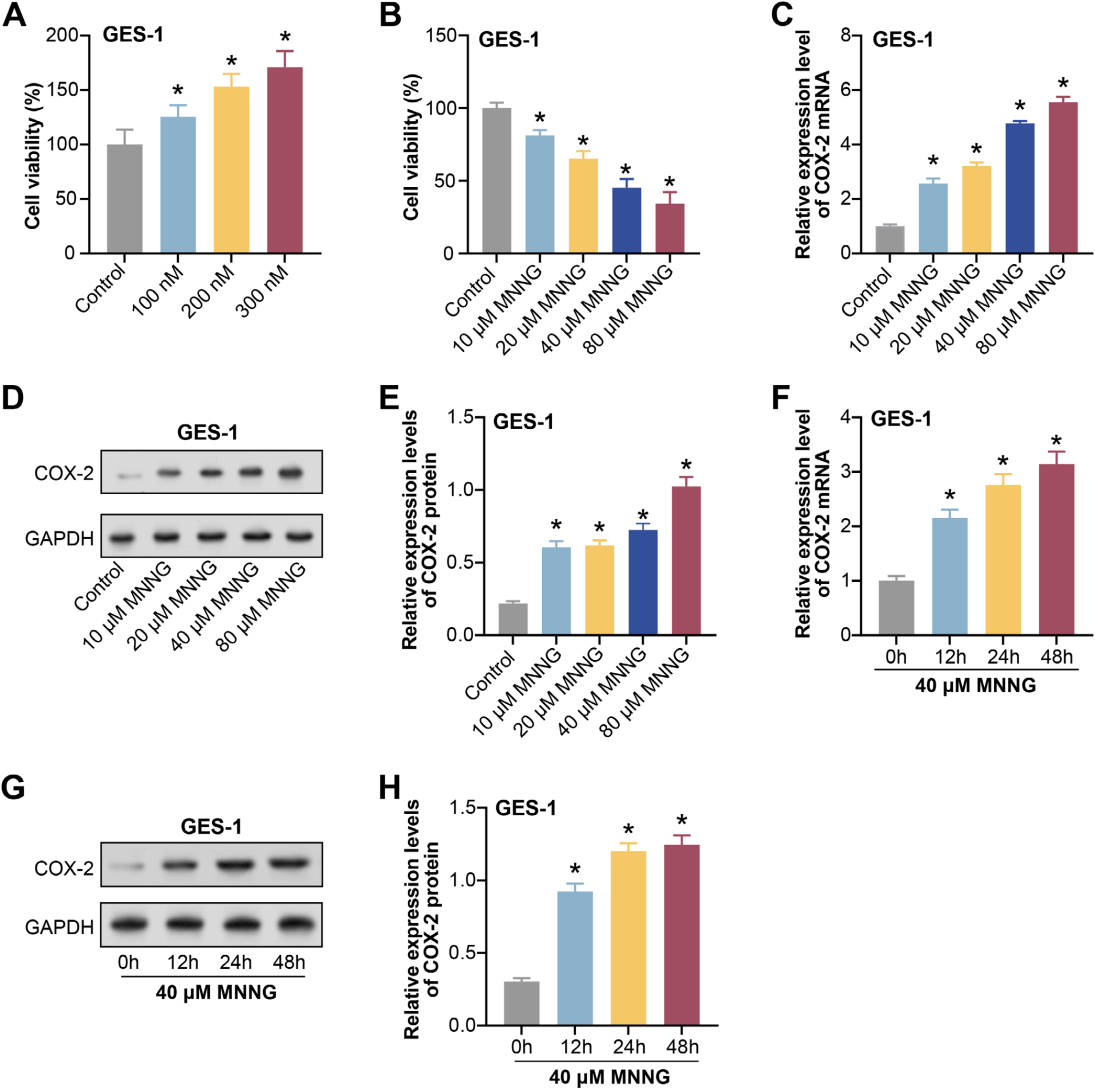
Effects of VC and MNNG induction on GES‐1 cell viability and COX‐2 expression. (A) GES‐1 cells were treated with 100, 200 and 300 nM VC, respectively, and CCK‐8 observed changes in cell viability. (B) GES‐1 cells were cultured with different concentrations of MNNG (10, 20, 40 and 80 μM) for 24 h, and CCK‐8 detected cell viability. (C–E) qRT‐PCR and WB detect changes in the expression level of COX‐2 as the concentration of MNNG increases. (F–H) GES‐1 cells were treated with 40 μM MNNG for 0, 12, 24 and 48 h, respectively. qRT‐PCR and WB were used to detect the expression level of COX‐2. CCK‐8, cell counting kit‐8; MNNG, N‐methyl‐N′‐nitro‐N‐nitrosoguanidine; qRT‐PCR, quantitative real‐time polymerase chain reaction; VC, vitamin C; WB, western blot. *p < 0.05.

Through pre‐experiments, we evaluated the effects of different VC concentrations on cell viability, apoptosis and oxidative stress responses. We determined that 100, 200 and 300 nM concentrations could significantly affect cell behaviour without inducing toxicity. GES‐1 cells were exposed to varying VC concentrations (100, 200 and 300 nM), and the changes in cell viability were observed by CCK‐8 assay (Figure 6A). We refer to the study by Li et al. Specific MNNG concentrations are effective in inducing fine CAG [19]. These findings provided a crucial experimental basis that helped us determine the concentration of MNNG used in our experiments. GES‐1 cells were cultured with different concentrations of MNNG (10, 20, 40 and 80 μM) for 24 h. The CCK‐8 experiment demonstrated that as MNNG concentrations increased, cell viability dramatically dropped (Figure 6B). COX‐2 can be used as an inflammatory marker. qRT‐PCR and WB analysis have shown that COX‐2 expression levels gradually increased with increasing MNNG concentrations (Figure 6C–E). Subsequently, GES‐1 cells received a treatment of 40 μM MNNG for 0, 12, 24 and 48 h. COX‐2 expression levels grew gradually with an extended induction period, as demonstrated by qRT‐PCR and WB analysis (Figure 6F–H).

### 3.7 VC Inhibits MNNG‐Induced Apoptosis in GES‐1 Cells

**FIGURE 7:**
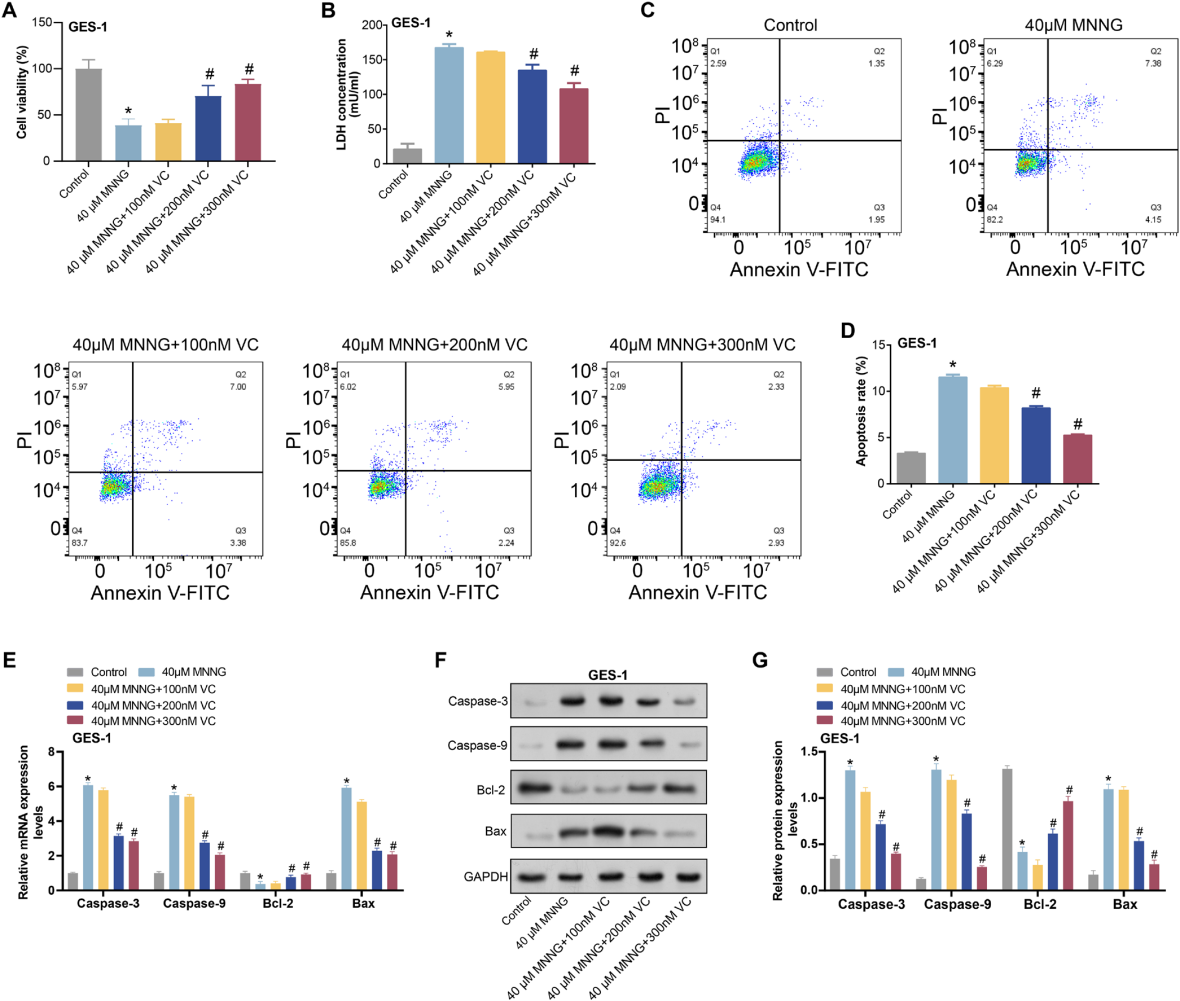
Protective effect of VC on MNNG‐induced cytotoxicity and apoptosis in GES‐1 cells. (A) CCK‐8 detects the viability of GES‐1 cells in different groups. Groups include: Control; 40 μM MNNG; 40 μM MNNG + 100 nM VC; 40 μM MNNG + 200 nM VC; 40 μM MNNG + 300 nM VC. (B) LDH kit detects LDH levels in GES‐1 cells under different conditions. Groups include: Control; 40 μM MNNG; 40 μM MNNG + 100 nM VC; 40 μM MNNG + 200 nM VC; 40 μM MNNG + 300 nM VC. (C and D) Flow cytometry detects the apoptosis of GES‐1 cells under different conditions. Groups include: Control; 40 μM MNNG; 40 μM MNNG + 100 nM VC; 40 μM MNNG + 200 nM VC; 40 μM MNNG + 300 nM VC. (E–G) qRT‐PCR and WB detected the expression of apoptotic markers Caspase‐3, Caspase‐9, Bcl‐2 and Bax in GES‐1 cells. Groups include: Control; 40 μM MNNG; 40 μM MNNG + 100 nM VC; 40 μM MNNG + 200 nM VC; 40 μM MNNG + 300 nM VC. CCK‐8, cell counting kit‐8; LDH, lactate dehydrogenase; MNNG, N‐methyl‐N′‐nitro‐N‐nitrosoguanidine; qRT‐PCR, quantitative real‐time polymerase chain reaction; VC, vitamin C; WB, western blot. *p < 0.05 vs control, ^#^ *p* < 0.05 vs. 40 μM MNNG.

CCK‐8 assay revealed a significant decrease in GES‐1 cell viability after 24 h of treatment with 40 μM MNNG. Subsequently, supplementation with 100, 200 and 300 nM of VC for an additional 24 h following MNNG induction showed that 200 and 300 nM of VC significantly attenuated the MNNG‐induced decline in cell viability (Figure 7A). Furthermore, the LDH assay demonstrated a significant increase in LDH levels following treatment with 40 μM MNNG, which was mitigated by supplementation with 200 and 300 nM VC (Figure 7B). Similarly, after being treated with 40 μM MNNG, flow cytometry analysis showed a considerable increase in apoptosis, which was attenuated by supplementation with 200 and 300 nM VC, particularly at 300 nM (Figure 7C,D). Subsequent qRT‐PCR and WB analysis of apoptosis‐related proteins Caspase‐3, Caspase‐9, Bcl‐2 and Bax in GES‐1 cells showed that proapoptotic proteins were significantly upregulated and anti‐apoptotic proteins were significantly downregulated by treatment with 40 μM MNNG, which was alleviated by supplementation with 200 and 300 nM VC, especially at 300 nM (Figure 7E–G). These results suggest that VC attenuates MNNG‐induced GES‐1 cytotoxicity and apoptosis by increasing cell viability, reducing LDH release and decreasing the expression of proapoptosis‐related proteins.

### 3.8 Knockdown of IGFBP7 Inhibits Proliferation and Promotes Apoptosis of GSE‐1 Cells

**FIGURE 8:**
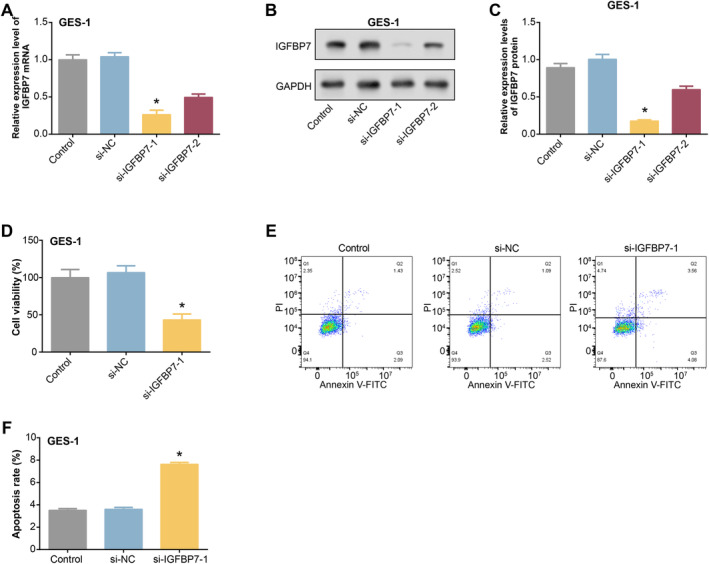
Effects on GES‐1 cell phenotype after knocking down *IGFBP7*. (A–C) QRT‐PCR and WB detected the knockdown efficiency of *si‐IGFBP7‐1* and *si‐IGFBP7‐2*. (D) CCK‐8 measured the cell viability of GES‐1 cells induced by *si‐IGFBP7‐1*. (E and F) The apoptosis of GES‐1 cells induced by si‐*IGFBP7*‐1 was detected by flow cytometry. CCK‐8, cell counting kit‐8; qRT‐PCR, quantitative real‐time polymerase chain reaction; WB, western blot. *p < 0.05.

GES‐1 cells were transfected with siRNAs and si‐NC targeting *IGFBP7*, and qRT‐PCR and WB analysis showed that *si‐IGFBP7‐1* showed higher efficiency in reducing *IGFBP7* mRNA and protein levels and was selected as the siRNA for subsequent experiments (Figure 8A–C). CCK‐8 assay showed that induction of GES‐1 cells with *si‐IGFBP7‐1* significantly decreased cell viability (Figure 8D). Furthermore, flow cytometry analysis revealed that the knockdown of *IGFBP7* in GSE‐1 considerably increased cell apoptosis (Figure 8E,F). These results demonstrate the critical function of *IGFBP7* in controlling the viability and apoptosis of GES‐1 cells.

### 3.9 MNNG and VC Regulate IGFBP7 Expression in GES‐1 Cells

**FIGURE 9:**
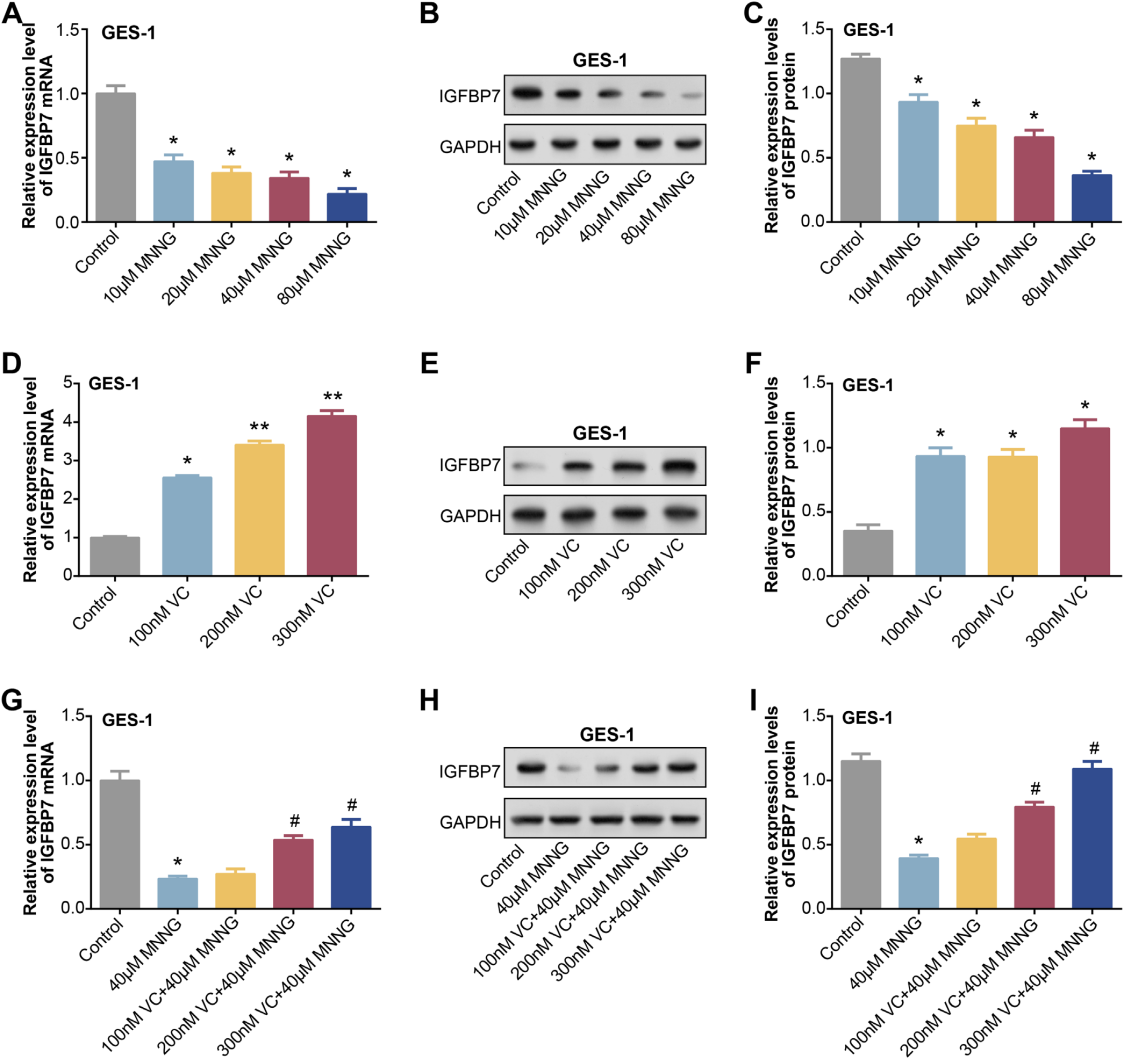
MNNG and VC regulate *IGFBP7* expression in GES‐1 cells. (A–C) After different concentrations of MNNG (10, 20, 40 and 80 μM) were treated with GES‐1 cells for 24 h, the expression level of *IGFBP7* was detected by qRT‐PCR and WB. (D–F) GES‐1 cells were induced by different concentrations of VC (100, 200 and 300 nM), and qRT‐PCR and WB detected the expression level of IGFBP7. (G–I) qRT‐PCR and WB were used to detect the expression of IGFBP7 in GES‐1 cells under different states. Groups include: Control; 40 μM MNNG; 40 μM MNNG + 100 nM VC; 40 μM MNNG + 200 nM VC; 40 μM MNNG + 300 nM VC. MNNG, N‐methyl‐N′‐nitro‐N‐nitrosoguanidine; qRT‐PCR, Quantitative real‐time polymerase chain reaction; VC, vitamin C; WB, western blot. *p < 0.05 vs control, ^#^ *p* < 0.05 vs 40 μM MNNG.

Following a 24‐h treatment with varying doses of MNNG (10, 20, 40 and 80 μM), *IGFBP7* expression levels were assessed by qRT‐PCR and WB analysis (Figure 9A–C). The results indicated a gradual decrease in *IGFBP7* expression levels with increasing concentrations of MNNG. Furthermore, GES‐1 cells were induced with varying concentrations of VC (100, 200 and 300 nM). It was observed that as the concentration of VC increased, the expression levels of *IGFBP7* also gradually increased (Figure 9D–F). Additionally, qRT‐PCR and WB analysis demonstrated that treatment with 40 μM MNNG significantly decreased *IGFBP7* expression levels, mitigated by adding VC (Figure 9G–I). These findings suggest a regulatory role of both MNNG and VC in modulating *IGFBP7* expression in GES‐1 cells.

### 3.10 Effects of Combined VC and IGFBP7 Knockdown on MNNG‐Induced Proliferation and Apoptosis of GES‐1 Cells

**FIGURE 10:**
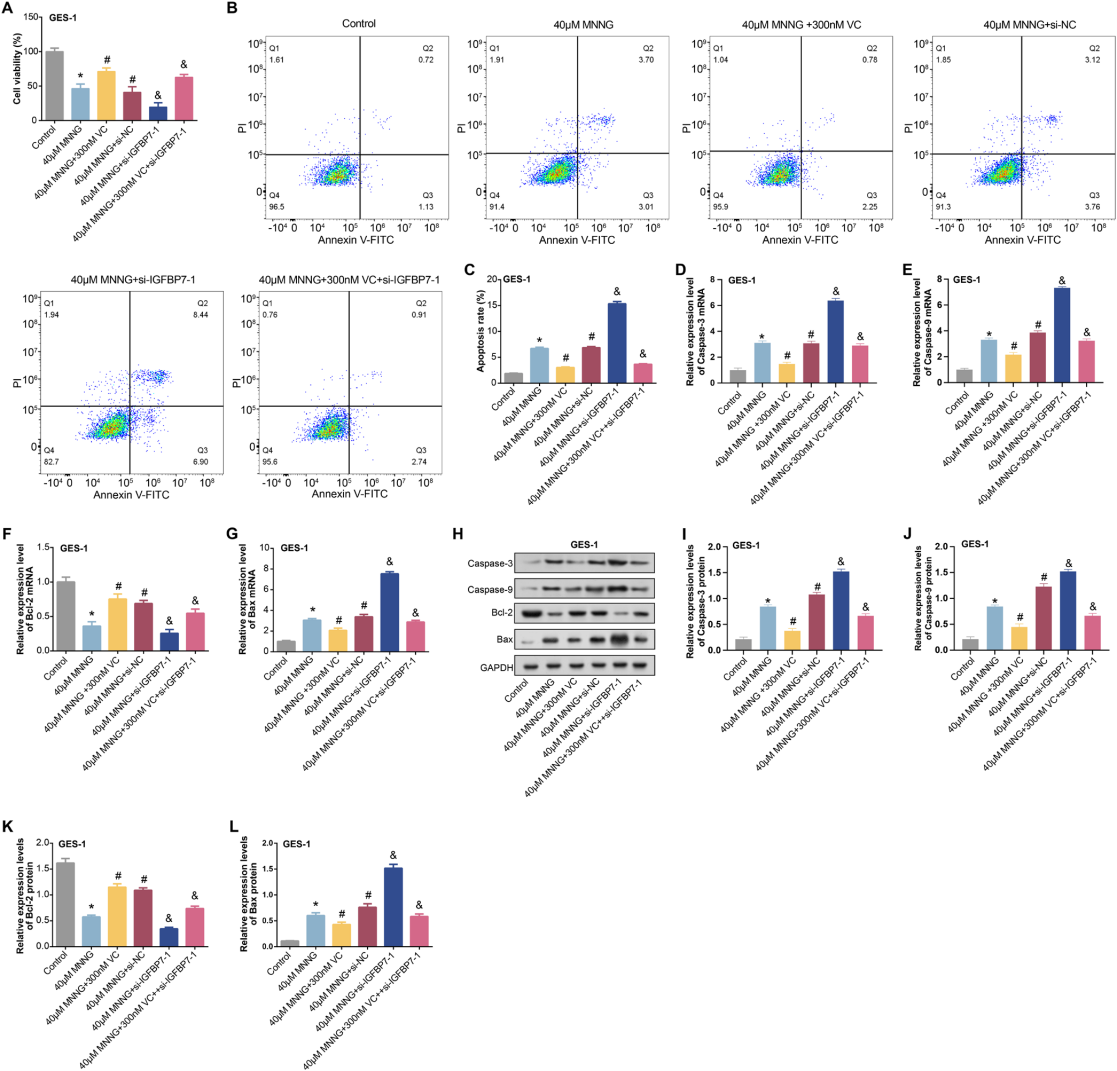
MNNG, VC and *IGFBP7* regulate cell viability and apoptosis in GES‐1 cells. (A) CCK‐8 detected the activity of GES‐1 cells under different induction. The groups are as follows: Control; 40 μM MNNG; 40 μM MNNG + 300 nM VC; 40 μM MNNG + si‐NC; 40 μM MNNG + si‐*IGFBP7*‐1; 40 μM MNNG + 300 nM VC + si‐*IGFBP7*‐1. (B and C) Flow cytometry was used to detect the apoptosis of GES‐1 cells under different induction. The groups are as follows: Control; 40 μM MNNG; 40 μM MNNG + 300 nM VC; 40 μM MNNG + si‐NC; 40 μM MNNG + si‐*IGFBP7*‐1; 40 μM MNNG + 300 nM VC + si‐*IGFBP7*‐1. (D–L) qRT‐PCR and WB were used to detect the expression of Caspase‐3, Caspase‐9, Bcl‐2 and Bax in GES‐1 cells under different induction. The groups are as follows: Control; 40 μM MNNG; 40 μM MNNG + 300 nM VC; 40 μM MNNG + si‐NC; 40 μM MNNG + si‐*IGFBP7*‐1; 40 μM MNNG + 300 nM VC + si‐*IGFBP7*‐1. CCK‐8, cell counting kit‐8; MNNG, N‐methyl‐N′‐nitro‐N‐nitrosoguanidine; qRT‐PCR, quantitative real‐time polymerase chain reaction; VC, vitamin C; WB, western blot. *p < 0.05 vs control, ^#^ *p* < 0.05 vs. 40 μM MNNG, ^&^ *p* < 0.05 vs 40 μM MNNG + 300 nM VC.

GES‐1 cells were treated with 40 μM MNNG, 300 nM VC and *si‐IGFBP7‐1*. CCK‐8 assay indicated a significant decrease in cell viability following a 24‐h 40 μM MNNG treatment in GES‐1 cells, which was mitigated by adding 300 nM VC. Further induction of cells with si‐IGFBP7‐1 upon treatment with 40 μM MNNG resulted in a more pronounced decrease in cell viability, which was reversed by adding VC (Figure 10A). However, flow cytometry analysis showed contrasting results (Figure 10B,C). MNNG treatment significantly increased cell apoptosis, while VC treatment reduced cell apoptosis. *IGFBP7* knockdown further increased apoptosis in MNNG‐treated cells. Compared with *IGFBP7* knockdown, VC and *IGFBP7* knockdown combined treatment reduced apoptosis, indicating a protective effect of VC (Figure 10B,C). qRT‐PCR and WB analysis were performed to examine the expression of Caspase‐3, Caspase‐9, Bcl‐2 and Bax in GES‐1 cells. MNNG treatment decreased Bcl‐2 expression and increased the expression of Caspase‐3, Caspase‐9 and Bax, indicating enhanced apoptosis, which was partially reversed by VC treatment, while IGFBP7 knockdown further enhanced the proapoptotic effect of MNNG. Notably, the combined treatment of VC and IGFBP7 knockdown partially restored MNNG‐induced apoptosis (Figure 10D–10L). These findings shed light on the interplay between MNNG, VC and *IGFBP7* in regulating cell viability and apoptosis in GES‐1 cells.

### 3.11 Effects of VC and MNNG on HIF‐1α/VEGF Signalling Pathway Protein Expression

**FIGURE 11:**
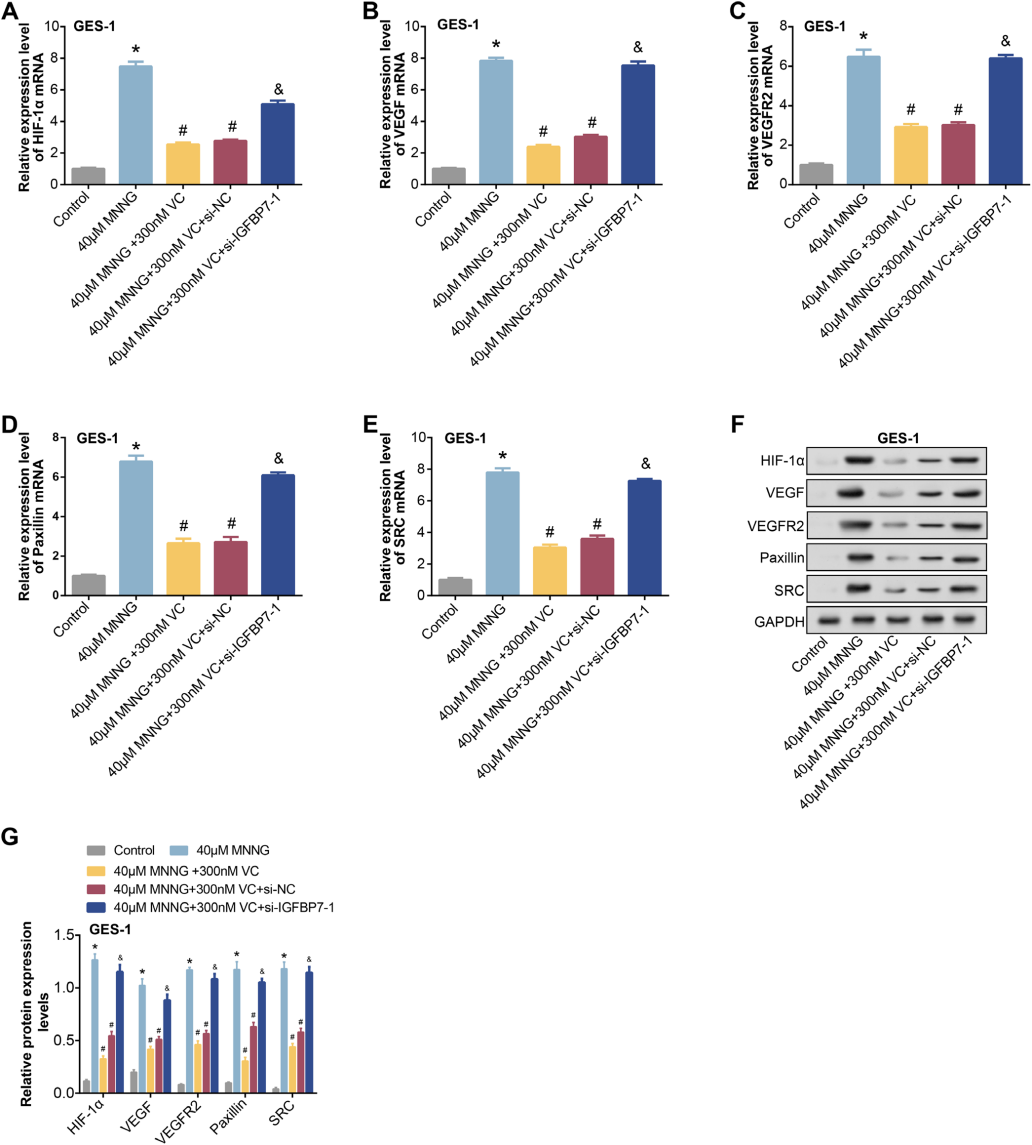
Effects of VC and MNNG on HIF‐1α/VEGF signalling pathway protein expression [14]. (A–G), qRT‐PCR and WB were used to detect the expression of HIF‐1α, VEGF, VEGFR2, Paxillin and SRC in GES‐1 cells under different induction. The groups are as follows: Control; 40 μM MNNG; 40 μM MNNG + 300 nM VC; 40 μM MNNG + 300 nM VC + si‐NC; 40 μM MNNG + 300 nM VC + si‐*IGFBP7*‐1. MNNG, N‐methyl‐N′‐nitro‐N‐nitrosoguanidine; qRT‐PCR, quantitative real‐time polymerase chain reaction; VC, vitamin C; WB, western blot. *p < 0.05 vs control, ^#^ *p* < 0.05 vs 40 μM MNNG, ^&^ *p* < 0.05 vs 40 μM MNNG + 300 nM VC.

The expression levels of proteins associated with the HIF‐1α/VEGF signalling pathway, comprising HIF‐1α, VEGF, VEGFR2, Paxillin and SRC, were assessed using qRT‐PCR and WB analysis (Figure 11A–G). The outcomes revealed a notable rise in the expression of HIF‐1α, VEGF, VEGFR2, Paxillin and SRC after 24 h of treatment with 40 μM MNNG in GES‐1 cells. The addition of 300 nM VC attenuated this upregulation. Furthermore, cotreatment with 40 μM MNNG and 300 nM VC, followed by induction with *si‐IGFBP7‐1*, resulted in a significant increase in the expression of HIF‐1α, VEGF, VEGFR2, Paxillin and SRC. However, the levels did not reach those induced by 40 μM MNNG alone. These findings suggest a complex interplay between MNNG, VC and *IGFBP7* in modulating the expression of proteins involved in the HIF‐1α/VEGF signalling pathway in GES‐1 cells.

### 3.12 VC and *IGFBP7* Affect MNNG‐Induced Chronic Atrophic Gastritis by Regulating the HIF‐1α/VEGF Signalling Pathway

**FIGURE 12:**
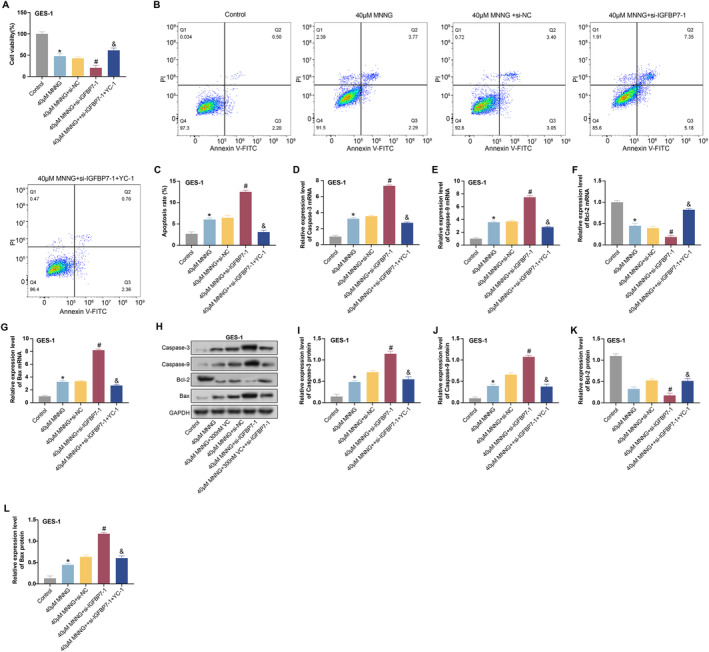
MNNG, *IGFBP7* and YC‐1 regulate the viability and apoptosis of GES‐1 cells. (A) CCK‐8 assay for the activity of GES‐1 cells under different induction conditions. The groups were as follows: Control; 40 μM MNNG; 40 μM MNNG + si‐NC; 40 μM MNNG + *si‐IGFBP7‐1*; 40 μM MNNG + *si‐IGFBP7‐1* + YC‐1. (B and C) Flow cytometry to detect apoptosis of GES‐1 cells under different induction conditions. The groups were as follows: Control; 40 μM MNNG; 40 μM MNNG + si‐NC; 40 μM MNNG + *si‐IGFBP7‐1*; 40 μM MNNG + *si‐IGFBP7‐1* + YC‐1. (D–L) The expression of Caspase‐3, Caspase‐9, Bcl‐2 and Bax in GES‐1 cells under different induction conditions was detected by qRT‐PCR and WB. The groups were as follows: Control; 40 μM MNNG; 40 μM MNNG + si‐NC; 40 μM MNNG + *si‐IGFBP7‐1*; 40 μM MNNG + *si‐IGFBP7‐1* + YC‐1.CCK‐8, cell counting kit‐8; MNNG, N‐methyl‐N′‐nitro‐N‐nitrosoguanidine; qRT‐PCR, quantitative real‐time polymerase chain reaction; WB, western blot. Compared with control, *p < 0.05; compared with 40 μM MNNG, ^#^ *p* < 0.05; compared with 40 μM MNNG + si‐IGFBP7‐1, ^&^ *p* < 0.05.

In experiments exploring the effects of VC and MNNG on the HIF‐1α/VEGF signalling pathway, we treated GES‐1 cells with a combination of 40 μM MNNG, *si‐IGFBP7‐1* and YC‐1, a known HIF‐1α inhibitor. The effect of cell viability was assessed by CCK‐8 assay. The results showed that the combination treatment of MNNG with *si‐IGFBP7‐1* significantly inhibited the viability of GES‐1 cells, while the addition of YC‐1 significantly reversed this inhibitory effect (Figure 12A). This finding suggests that YC‐1 may have a protective effect against cellular stress and decreased viability induced by anti‐MNNG and *si‐IGFBP7‐1*. Flow cytometry analysis further revealed changes in apoptosis. Contrary to the results of CCK‐8 experiments, the cotreatment of MNNG and *si‐IGFBP7‐1* significantly increased apoptosis, whereas the treatment of YC‐1 decreased apoptosis (Figure 12B,C). These results imply that YC‐1 may protect cells by inhibiting apoptosis, which may be related to regulating the HIF‐1α/VEGF signalling pathway. To understand the molecular mechanisms behind these changes, we analysed the expression of Caspase‐3, Caspase‐9, Bcl‐2 and Bax by qRT‐PCR and WB. Cotreatment of MNNG and *si‐IGFBP7‐1* decreased the expression of Bcl‐2 and increased Caspase‐3, Caspase‐9 and Bax expression, suggesting that apoptosis was enhanced (Figure 12D–L). Remarkably, treatment with YC‐1 partially reversed these changes, inhibiting apoptosis. These results emphasise the potential role of VC and *IGFBP7* in regulating the HIF‐1α/VEGF signalling pathway, especially in the context of MNNG‐induced cellular stress and apoptosis. These effects of VC and *IGFBP7* may be related to their modulation of the HIF‐1α/VEGF signalling pathway, which could have significant implications.

## 4 Discussion

WGCNA is a powerful bioinformatics tool used to explore relationships between genes in several samples. WGCNA allows researchers to uncover underlying biological processes and potential biomarkers by identifying key modules connected to specific characteristics or circumstances. Zheng et al. found through WGCNA that CD14 and CSF1R showed high expression levels in osteoarthritis [6] and gastritis, highlighting CD14 and CSF1R as promising therapeutic targets for treating OA and gastritis [20]. Similarly, Qin et al. utilised WGCNA to identify hub genes linked to GC progression, including *GAGE12J*, *PHGR1*, *POLG2* and *COL21A1* [21]. These genes, modulated in gastritis and GC, influence tumorigenesis‐related pathways such as DNA repair and KRAS signalling, shedding light on GC pathogenesis. Additionally, Jia et al. demonstrated that WGCNA unveils staged characteristics of gastritis–cancer transformation, revealing distinct expression patterns at different stages [22]. The study also pinpointed potential biomarkers for atrophic gastritis and GC, providing valuable insights into disease progression and diagnostic strategies. In this study, we identified the key module, the blue module, from the GSE153224 dataset using WGCNA. Subsequently, we performed a cross‐analysis of upregulated DEGs, downregulated DEGs and genes in the blue module from GSE153224 to obtain overlapping genes. These overlapping genes were then subjected to PPI network analysis, followed by a cross‐analysis of the top 10 genes from each of the three topological analyses, identifying six overlapping genes. Finally, we analysed the expression of these six key overlapping genes in the GSE153224 and GSE27411 datasets, among which only *IGFBP7* was significantly underexpressed in CAG samples, thus identifying *IGFBP7* as a hub gene for further analysis.

MNNG is a chemical compound frequently utilised in laboratory research settings to induce the development of malignant cells [23]. In the context of CAG, MNNG is commonly employed to model the pathological conditions associated with gastric inflammation and epithelial cell damage, reflecting the underlying molecular mechanisms of CAG progression [24]. Xie et al. discovered that treating MNNG‐induced gastritis and dysplasia with Wei‐fu‐chun tablet (WFC) inhibits inflammation via NF‐κB pathway modulation [25]. WFC improves histopathology, reduces inflammation markers and suppresses *CDX2* expression, halting gastric lesion progression. Similarly, Wu et al. found that Granule Dendrobii [26] effectively alleviates MNNG‐induced CAG in rats [27]. GD enhances gastric mucosa histology, reduces inflammation, reverses gastric atrophy and intestinal metaplasia and boosts haemoglobin levels. Furthermore, Wang et al. explored Jian‐Pi‐Yi‐Qi‐Fang's (JPYQF) mechanism in treating CAG induced by MNNG [28]. JPYQF enhances body weight, mitigates gastric atrophy and inflammation and upregulates markers associated with gastric stem cell proliferation, differentiation and Wnt signalling, highlighting its promise in CAG therapy. The above studies indicate that by inducing CAG‐like changes in cell lines or animal models, MNNG becomes a valuable tool for studying the pathogenesis of CAG and potential therapeutic interventions.

As a potent antioxidant, VC neutralises free radicals and protects cells from oxidative damage [29]. This antioxidant property is vital for the defence and managing various inflammatory diseases, including gastritis. Our study showed that VC treatment significantly increased cell viability and reduced apoptosis in GES‐1 cells. VC also positively regulated various blood parameters in CAG patients, especially reducing G‐17 and IL‐6 levels and increasing the PGI/PGII ratio, highlighting its anti‐inflammatory effects. An enzyme called COX‐2 is linked to pain and inflammation. In our study, GES‐1 cell viability increased significantly with increasing VC concentrations, while cell viability decreased with increasing MNNG concentrations. In addition, COX‐2 expression in GES‐1 cells gradually increased with increasing MNNG concentrations and treatment time. These findings highlight the effects of VC and MNNG on GES‐1 cell viability and COX‐2 expression, underscoring their potential role in regulating cellular responses to oxidative stress and genotoxic insults. Therefore, incorporating VC into the treatment regimen for CAG could provide significant therapeutic benefits, leveraging its multifaceted biological activities to improve patient outcomes.

Cells undergo apoptosis or programmed cell death to remove damaged cells and preserve tissue homoeostasis [30]. Apoptosis dysregulation has been linked to several illnesses, such as autoimmune diseases, cancer and neurodegenerative diseases [31]. Previous studies have highlighted different mechanisms of apoptosis. Zhang et al. discovered that while ginsenoside Rg3 reduces MNNG‐induced DNA damage and apoptosis in normal human cells, it causes damage to DNA in human osteosarcoma cells [32]. Yu et al. discovered that the activation of poly(ADP‐ribose) polymerase‐1 (PARP‐1) mediates cell death via apoptosis‐inducing factor (AIF) translocation, with MNNG, H_2_O_2_ and N‐methyl‐d‐aspartate inducing AIF translocation and cell death through PARP‐1 activation, suggesting a route of programmed cell death that is independent of caspase [33]. Additionally, Yan et al. revealed that MNNG induces damage and apoptosis in GES‐1 cells, altering morphology and blocking cell‐cycle progression, with upregulated expressions of β‐catenin, *MMP7*, c‐Met and GSK‐3β, suggesting the involvement of the Wnt/β‐catenin pathway in cell injury mechanisms [34]. Our study demonstrates that MNNG treatment reduces GES‐1 cell viability, whereas VC significantly attenuates the decrease in MNNG‐induced viability. LDH assay indicated a significant increase in LDH levels post‐MNNG treatment, which VC alleviated. After MNNG administration, flow cytometry analysis showed a substantial increase in cell death, which VC attenuated, especially at 300 nM. In addition, qRT‐PCR and WB analyses showed that MNNG treatment increased the expression of proapoptotic proteins caspase‐3, caspase‐9 and Bax and decreased the expression of anti‐apoptotic protein Bcl‐2 in GES‐1 cells, which VC alleviated. This suggests that VC can mitigate MNNG‐induced damage and apoptosis in GES‐1 cells.

Prior research has underscored the function of *IGFBP7* in a multitude of conditions. Zang et al. demonstrated that Eleutheroside B mitigates acute kidney injury [9] by inhibiting inflammation and apoptosis, reducing *IGFBP7* expression and activating the IGF pathway to promote cell proliferation [35]. Similarly, Yang et al. demonstrated that by preventing *IGFBP7*/IGF1R‐mediated inflammation and programmed cell death, as well as by decreasing renal necroptosis through the RIPK1/RIPK3/MLKL pathway, genocide XLIX protects against AKI [14]. Xu et al. demonstrated that *IGFBP7* exacerbates sepsis‐induced acute lung injury by inducing apoptosis and cytotoxicity in pulmonary microvascular endothelial cells and activating the ERK1/2 pathway during sepsis‐induced inflammation [36]. In light of these findings, we want to examine the function of *IGFBP7* in CAG‐related cell apoptosis and inflammation. Our study demonstrated that si‐*IGFBP7*‐1 markedly enhanced apoptosis in GES‐1 cells. qRT‐PCR and WB analysis indicated a negative correlation between MNNG concentration and *IGFBP7* expression, while VC showed a positive correlation. VC attenuated the MNNG‐induced decrease in *IGFBP7* expression. MNNG significantly increased apoptosis in GES‐1 cells, which VC counteracted. Additional induction with si‐*IGFBP7*‐1 in MNNG‐treated cells resulted in a more pronounced increase in apoptosis, which VC reversed. These findings indicate that VC mitigates MNNG‐induced *IGFBP7* downregulation and apoptosis in GES‐1 cells.

The HIF‐1α/VEGF signalling pathway is crucial for angiogenesis, particularly under hypoxic conditions [26]. A crucial transcription factor is HIF‐1α which regulates VEGF expression, a potent angiogenic factor [37]. Under hypoxia, HIF‐1α accumulates and translocates to the nucleus, where it binds to hypoxia‐responsive elements (HREs) in the promoter regions of target genes, including *VEGF* [38]. This upregulates VEGF expression, which then binds to its receptor *VEGFR2* on endothelial cells, initiating signalling cascades that promote proliferation, migration and survival. Activation of *VEGFR2* triggers various intracellular signalling pathways, ultimately leading to angiogenesis [39]. Paxillin, an adaptor protein, is essential for integrin‐mediated migration and cell attachment by linking integrins to intracellular signalling molecules [40]. Nonreceptor tyrosine kinase SRC kinase participates in several signalling pathways that control cell migration, survival and proliferation [41]. In the context of angiogenesis, Paxillin acts downstream of *VEGFR2* and is involved in *VEGF*‐induced endothelial cell migration and invasion [42]. *SRC* kinase is also activated downstream of *VEGFR2* and regulates endothelial cell behaviour during angiogenesis [43]. Overall, *HIF‐1α* regulates *VEGF* expression, which activates *VEGFR2* signalling, leading to downstream activation of *Paxillin* and *SRC*, ultimately promoting angiogenesis.

In studies by Yin et al., early stages of CAG induction exhibited reduced gastric mucosal blood flow alongside increased mRNA expression of *COX‐2*, *HIF‐1α*, *VEGFR1* and *VEGFR2* [44]. Significant elevations in *HIF‐1α*, *COX‐2* and *VEGFR2* levels post‐CAG induction suggest COX‐2/HIF‐1α/VEGF pathway activation during CAG development. Another study by Yin et al. found that Weiqi Decoction (WQD) treatment mitigated CAG‐induced changes in gastric mucosa by restoring microcirculation and inhibiting *HIF‐1α*, *COX‐2*, *VEGFR1* and *VEGFR2* expression [45]. Additionally, Wang K et al. found that acupoint catgut embedding reduced gastric mucosal injury in CAG rats by downregulating HIF‐1α and VEGF protein expression [46]. These studies suggest that targeting the HIF‐1α/VEGF pathway possibly offers therapeutic benefits in CAG. In our study, treatment with MNNG enhanced the expression of HIF‐1α, VEGF, SRC, Paxillin and VEGFR2 genes and proteins. Pretreatment with VC deactivated the HIF‐1α‐mediated VEGF signalling pathway and inhibited angiogenesis by reducing the expression of these genes and proteins. Additionally, the addition of *IGFBP7* knockdown attenuated this inhibition. YC‐1 treatment was able to partially counteract the adverse effects of MNNG cotreatment with *si‐IGFBP7‐1* on GES‐1 cells. These findings reveal the potential therapeutic role of interventions targeting the HIF‐1α/VEGF pathway in MNNG‐induced CAG.

Existing CAG treatments focus on eradicating *H. pylori* infection, relief of symptoms and preventing complications such as gastric ulcers and malignancies [47]. These treatments have limited efficacy in reversing gastric mucosal atrophy and improving long‐term prognosis. Our study found that VC significantly increased GES‐1 cell viability, reduced apoptosis and showed anti‐inflammatory effects by modulating several blood parameters, especially decreasing G‐17 and IL‐6 levels and increasing PGI/PGII ratio in CAG patients. In addition, the downregulation of *IGFBP7* further affected these effects. These results suggest that VC‐ and *IGFBP7*‐targeting strategies may offer a new therapeutic avenue to improve gastric mucosal health and reduce inflammation, which may be more effective than conventional treatments. Conventional CAG treatments, such as antibiotics and acid‐suppressing drugs, are usually considered safe in the short term. Still, long‐term use may result in side effects, such as antibiotic resistance and acid‐suppressing drug‐associated osteoporosis [48, 49]. As a water‐soluble vitamin, VC is usually considered safe and has few side effects at recommended doses [50]. However, high doses of VC may cause side effects such as diarrhoea and nausea. Targeted therapies for *IGFBP7* have less data on safety, and further studies are needed to assess their potential side effects and long‐term impact. Existing treatments for CAG, such as antibiotic therapy and acid‐suppressive therapy, are widely used in clinical practice and are feasible in resource‐rich areas. VC, a widely available and relatively low‐cost nutrient, is highly possible for CAG treatment, especially in resource‐limited settings. Therapeutic strategies targeting *IGFBP7* are still in the research phase, and more clinical trials are needed to assess their feasibility in real clinical settings.

Although our study reveals a potential role for VC and IGFBP7 in regulating the HIF‐1α/VEGF signalling pathway, we recognise that translating these findings into clinical practice will require overcoming several challenges. The experiments in this study were performed entirely under in vitro conditions, which may limit the direct clinical application of our results. In vitro experiments often do not fully replicate the complex physiological and pathological conditions found in vivo, and thus, our results need to be validated in in vivo models to confirm their relevance. We recognise possible differences between the in vitro experiments and the in vivo environment, including changes in cell behaviour, activation of signalling pathways and drug responsiveness. Challenges that may be encountered in translating in vitro findings into clinical practice include determining drug dose–effect relationships, pharmacokinetic and pharmacodynamic properties, and assessing long‐term therapy's safety and efficacy. For in vitro findings to have clinical applications, future studies must explore the mechanisms of action of VC and *IGFBP7* in vivo models and their potential in treating human diseases. We suggest that to validate these results in human studies, a series of preclinical and clinical studies, including dose‐ranging and toxicity studies in animal models, as well as preliminary clinical trials, are needed to assess the safety, tolerability and preliminary efficacy of VC and *IGFBP7* treatments. In addition, there is a need for large‐scale, randomised, controlled clinical trials to determine the effectiveness and applicability of these interventions in real‐world clinical settings. In considering high‐dose VC as a therapeutic strategy for CAG, we could not adequately discuss its potential side effects or challenges. The possible effects of long‐term VC administration require further study, including its impact on the gastrointestinal tract and other systems. Future studies should evaluate the safety and side effects of different doses of VC to determine the optimal treatment regimen.

## 5 Conclusions

This research elucidates the crucial responsibilities of VC and *IGFBP7* in alleviating MNNG‐induced CAG in GES‐1 cells. We analysed key gene modules and hub genes, especially *IGFBP7*, using WGCNA and DEGs. Our results showed that VC treatment significantly improved cell viability, reduced apoptosis and regulated inflammation and growth factors in patients with CAG. VC also attenuated MNNG‐induced cytotoxicity, apoptosis and COX‐2 expression while positively affecting *IGFBP7* expression. In addition, VC inactivated the HIF‐1α/VEGF signalling pathway, thereby reducing the expression of angiogenesis‐related proteins, while *IGFBP7* knockdown further enhanced these protective effects. These findings demonstrate the possible therapeutic benefits of VC and *IGFBP7* in managing CAG and provide insights into their mechanisms in regulating cell viability, apoptosis and inflammation.

## Author Contributions

**Xun Cheng:** conceptualization (equal), formal analysis (equal), writing – original draft (equal), writing – review and editing (equal). **Hao Gu:** conceptualization (equal), funding acquisition (equal), software (equal), writing – original draft (equal), writing – review and editing (equal). **Yulin Chong:** conceptualization (equal), formal analysis (equal), funding acquisition (equal), writing – original draft (equal), writing – review and editing (equal). **Fan Li:** conceptualization (equal), data curation (equal), resources (equal), writing – original draft (equal), writing – review and editing (equal). **Songhua Bei:** conceptualization (equal), methodology (equal), writing – original draft (equal). **Huanqing Li:** data curation (equal), formal analysis (equal), funding acquisition (equal), writing – original draft (equal), writing – review and editing (equal). **Jun Jiang:** conceptualization (equal), supervision (equal), validation (equal), writing – original draft (equal). **Ming Pan:** resources (equal), software (equal), supervision (equal), validation (equal), visualization (equal), writing – original draft (equal). **Li Feng:** investigation (equal), methodology (equal), project administration (equal), visualization (equal), writing – original draft (equal), writing – review and editing (equal). **Xiaohong Zhang:** conceptualization (equal), formal analysis (equal), software (equal), visualization (equal).

## Ethics Statement

Our study was conducted with the approval of the Shanghai Minhang Central Hospital Ethics Committee.

## Consent

The authors have nothing to report.

## Conflicts of Interest

The authors declare no conflicts of interest.

## Supporting information


**Figure S1.** Endoscopic images of twelve patients before and after 3 months of continuous VC administration. Gastroscopy images. The first 12 images are gastroscopy before VC treatment, and the last 12 are after VC treatment. VC: Vitamin C.

Table S1.

Table S2.

## Data Availability

The datasets used and analysed during the current study are available from the corresponding author upon reasonable request.
