# Supplementary figures and images for: Vitamin C Mediates IGFBP7 to Alleviate Chronic Atrophic Gastritis via the HIF‐1α/VEGF Pathway
Source: J Cell Mol Med. 2025 Feb 26;29(4):e70392. doi: 10.1111/jcmm.70392 (PMC11865351; doi:10.1111/jcmm.70392)

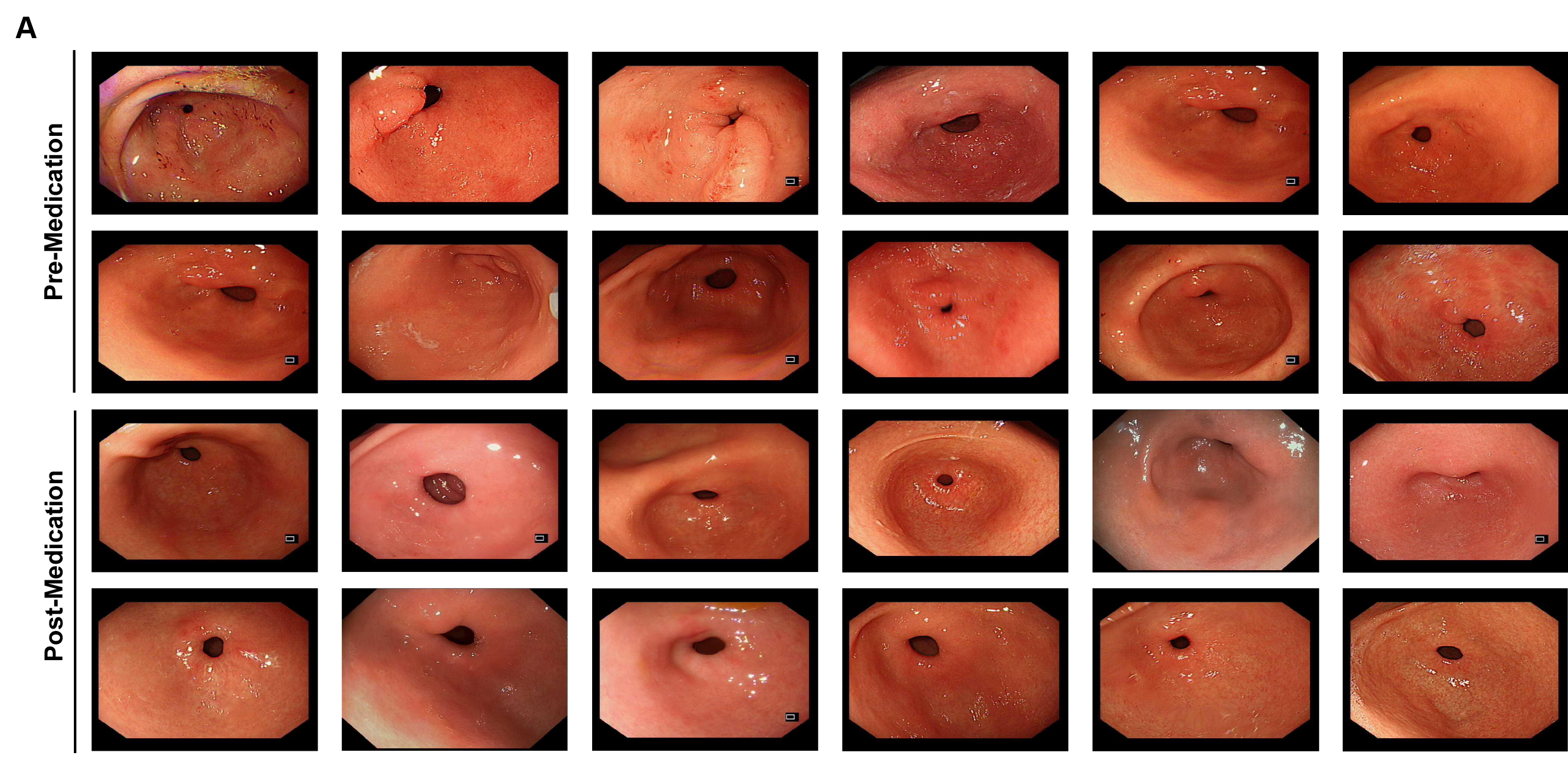

Supplement: Supplementary file 1 — Figure S1. Endoscopic images of twelve patients before and after 3 months of continuous VC administration. Gastroscopy images. The first 12 images are gastroscopy before VC treatment, and the last 12 are after VC treatment. VC: Vitamin C. [file JCMM-29-e70392-s001.tif]
